# ANNUAL WELLNESS SCREENING AND COVID-19: A MISSED OPPORTUNITY TO ADDRESS HEALTH DISPARITIES

**DOI:** 10.1093/geroni/igae098.1785

**Published:** 2024-12-31

**Authors:** Nancy Allen, Yao He, Norman Foster

**Affiliations:** University of Utah, Salt Lake City, Utah, United States; University of Utah, Salt Lake City, Utah, United States; University of Utah, Salt Lake City, Utah, United States

## Abstract

The COVID-19 pandemic had the greatest effect on the most vulnerable. Medicare annual wellness visits (AWV) are the best opportunity for early detection of cognitive impairment and could address inequities in dementia care. We examined how preventive care through AWV changed as our healthcare system responded to pandemic disruptions. We hypothesized that disruptions would exacerbate pre-existing disparities in AWV administration. We conducted an observational retrospective chart review of AWV of Medicare-eligible adults ≥65 years of age in our health system from Jan 2018 to Dec 2022. In 2020 at the beginning of the COVID-19 pandemic, there were 32.9% fewer AWV performed than in 2019. A month-by-month analysis showed a sharp 91% decline in AWV when clinic operations were most restricted. When operations largely resumed by June 2020, AWV returned to the pre-pandemic proportion of January 2020. However, previous disparities in AWV performance continued or were exacerbated. Post-pandemic, AWV were more frequent in younger (< 75 years; 32.5%) rather than older individuals (>75; 21.4%), in white (30.9%) rather than non-white patients (24.2%), and in non-Hispanic (30.5%) rather than Hispanic individuals (23.7%). Patients without diabetes had more AWV (31%) than those without (31% vs. 27.6%). Success in resumption of AWV occurred by the continuation of conscious and unconscious scheduling biases, rather than addressing previously recognized disparities. This represents a missed opportunity and highlights the need for policy changes to address health equity in AWV. The disparities in AWV mirror the disparities in dementia diagnosis and treatment.

